# The Influence of Constitutional Diathesis on the Development of Disease Germs

**Published:** 1883-02

**Authors:** 

**Affiliations:** Prof. of Surgery of the Paris Faculty


### LECTURE.—THE INFLUENCE OF CONSTITUTIONAL DIATHESIS ON THE DEVELOPMENT OF DISEASE GERMS.

*Gentlemen:* It seemed to be the general belief at one period, that it was but necessary for the microbe or morbid germ to enter the organism in order to become multiplied ad infinitum.

The presence of the parasitic germ and effraction of the tissues were regarded as the two conditions necessary, but always sufficient to determine infection and constitute the disease.

According to this accommodating theory, no matter what the constitutional condition of the individual, if the malignant charbon bacteride (bacillus) comes in contact with any point where the epidermis is excoriated, malignant pustule will of necessity be developed.

Many observations serve to show that this opinion is not well founded. The organism, in many cases, does not permit the entrance of the virus into the system, and the morbid germ languishes and perishes on sterile ground. Proofs of this assertion are not wanting; of those bitten by a rabid dog, all do not contract hydrophobia; certain individuals escape diphtheria, measles, variola and scarlatina, when exposed to the infection. There are some who do not contract gonorrhœa or syphilis from persons who have communicated the disease to others. I have among my patients a young lady, twenty-three years of age, who has been vaccinated nineteen times ineffectually. Such subjects are said to be refractory vis-ā-vis certain diseases.

On the other hand there are individuals who fall an easy prey to germs, septic or otherwise; if the infectious spores penetrate their tissues, disease is sure to follow. We have just mentioned malignant charbon; the flocks in the pastures of Normandy are hardly ever affected, while its ravages among animals in the neighboring provinces are well known. Finally, one or two successful vaccinations are generally considered sufficient; yet a physician in one of our hospitals contracted small-pox within less than a year after a successful inoculation.

It is evident that but a very slight change in the composition of the solids and fluids of the organism, is necessary to render the system refractory or not to the penetration of germs. What modifications do syphilis, variola, scarlatina, measles, and charbon, provoke in our tissues?

Chemistry cannot furnish a satisfactory reply; and yet, after a first attack, the organism has become improper for the nourishment a second time of the germs of these maladies, which attack but very exceptionally the same individual twice. There are no longer in the economy the same elements or the same quantity of these elements; the constituent principles are so altered that an organism, where certain germs formerly multiplied, no longer receives them.

A laboratory experience, reported by Duclaux in his remarkable work, demonstrates very strikingly the fragility of germs, their nutritive exigencies, and their extreme sensitiveness.

A culture liquid *(liquide de culture)* contains one-fifty-millionth part of zinc; one or two generations of aspergillus will completely absorb the metal, so as to render the existence of a new generation stunted or impossible. "In such a liquid a new planting, I was about to say a new inoculation, will remain without effect."

"If we add," continues M. Duclaux, "a sixteen-hundred-millionth part of nitrate of silver to the liquid of culture, vegetation is suddenly arrested. It will not even begin in a silver vase, although chemical investigation cannot demonstrate the presence of any part of the matter of the vase dissolved in the liquid of culture. If we suppose that the aspergillus were a human parasite, capable of living and undergoing development in the organism and invading it in all its parts, the quantity of nitrate of silver necessary to eradicate it from the body of a man weighing sixty kilograms would be forty milligrams. If it attacked only the blood, five milligrams would suffice to arrest the development of so sensitive an organism. It is true that other species may live where the aspergillus perishes. Each ferment has a special liquid which favors its development, whence the formula: "any one soil is not proper for all forms of culture; and a fertile spot is very soon worn out."

The passing or permanent modifications of the organism have then a direct influence on the evolution of the microbic disease. It is evident that certain conditions of the liquids, blood, lymph, etc., facilitate fermentations and cultures; and as the chemical composition of the liquid constituents of the organism vary from one malady to another, it may be advanced, that each constitutional disease, specific or not, renders the organism more apt to contract certain other maladies.

In other terms, the morbid germs find in the liquids of an organism, affected by a constitutional diathesis, a soil either propitious, indifferent, or refractory to their development.

Prof. Bouchard makes a similar declaration in his recent lectures: "among the agents of infection, there are certain species which prosper in the human organism, no matter whether it be in a state of health or diseased. There are other germs which respect the human subject when in a condition of health, and do not find in the solids or liquids of the organism a soil favorable for their development except under certain morbid conditions, which have induced a deterioration of the economy, in cases where alteration of nutrition has provoked chemical changes in the solids or liquids."

Such an affirmation is perhaps too general and sweeping, but we will seek to determine the conditions of the organism favorable to the development of certain germs, and obnoxious to others.

The humors or liquids of rheumatic, gouty, and diabetic subjects, charged with uric acid, the urates, or sugar (to mention only recognized chemical products), are very favorable to the development of the microbe of furuncle and anthrax; so much so that the appearance of these local affections should cause the observer to suspect the existence of the arthritic diathesis. It has long been known that anthrax is one of the most common complications of diabetes; but its frequency in gouty and rheumatic subjects and in those suffering from maladies due to defective nutrition *(ralentissement de la nutrition)* has not attracted sufficient attention.

On the contrary, the humors of scrofulous patients are so improper for the development of the furuncle germ, that a case of anthrax in a subject presenting scrofulous manifestations is yet to be reported.

This remarkable antagonism merits confirmation, and furnishes a very interesting subject for study. It must be observed, however, that the infecundity of scrofulous blood as regards furuncle germs is not absolute. Recently we observed a young girl, evidently consumptive, who presented around one knee an eruption of twenty-five furuncles. It is true that both the father and mother of this patient were subject to rheumatic attacks, and she had herself, from time to time, suffered from erratic pains in the joints; so that this case does not form an exception to the general law we have laid down.

M. Bouchard furnishes an example of two other parasites which do not prosper in the human subject, except in individuals affected with certain constitutional diseases, and under the influence of certain well-determined morbid conditions. "The microsporon furfur of ptyriasis versicolor has a marked predilection for phthisical and arthritic patients." As regards the oidium albicans, it is not developed, as is well known, except in debilitated subjects; during infancy in athrepsic children; in old age, in worn-out and feeble individuals, and at other periods of existence in patients worn out by a grave malady of long duration. Under such conditions the morbid product appears on surfaces bathed in acid mucous.

Veterinary medicine offers an analogous fact as regards scabies. Delafond and Bourguignon have demonstrated that healthy sheep, properly cared for, resist absolutely colonization by acari scabiei, while if these same sheep are poorly fed and become debilitated, they contract the disease very easily. If they are then again brought back to good condition, the disease disappears of itself. Could not we assimilate to these parasites those of gonorrhœa, described by Hallier in 1872, and later by Salisbury, Neisser, Bouchard, and Capitan? This disease would seem to be developed equally well in all subjects; but Ricord, when he gave his famous recipe for taking gonorrhœa, was perhaps wrong in accumulating all the external conditions which might aid in the development of the disease.

The condition of the individual about to be infected is of great consequence. Do we not observe cases where gonorrhœa first comes on with but slight cause, and, finding a fertile soil, becomes chronic and defies all the efforts of the physician? The flow may be arrested, but not completely disappear, and the patient is subject to a new attack on any change of diet, or after any form of excess.

Or, it is by no means an affair of chance, that certain individuals contract such grave and persistent forms of this disease; we are of opinion that the arthritic diathesis is the favorite soil for such luxuriant culture of infectious germs. We have observed many cases of interminable gonorrhœa, developed in individuals who presented all the signs of retarded or defective nutrition, and in such subjects exclusively. Attention to hygienic conditions, appropriate food, hydrotherapy and out-door exercise in the country, often alone sufficed to cure a gleet which had persisted for long periods, in spite of every form of injection and medication.

There is another microbe which has exactly the opposite tendency: I refer to the germ of tuberculosis, the existence of which appears to be fully demonstrated since Villemin proved the inoculability of the tuberculous granulation. This germ does not prosper or spread in the arthritic organism, while it invades and destroys with extreme energy the scrofulous subject. I had long been a unicist, believing in the identity of the scrofulous and tuberculous diathesis. I considered tubercle as forming the most advanced anatomical manifestation of the strumous diathesis, holding the same relation to scrofula as gumma to syphilis, or cancer to arthritism. But many objections to this view presented themselves, such as the coincidence of tuberculosis with cancer, the appearance, rare, it is true, of tuberculosis in subjects who had never presented the slightest trace of scrofula, and finally, the hereditary transmission of scrofula and the evident contagious nature of tuberculosis.

To-day I am of opinion that scrofula and tuberculosis are two distinct morbid conditions; the first due to some defect of nutrition, the second to parasitic invasion. Substituting for the notion of identity that of extreme affinity, the apparent contradictions between the results of clinical observation and those of experimentation, are almost entirely effaced.

It was formerly admitted that a certain degree of antagonism existed between malaria and tuberculosis; to-day opinions on the subject are divided. It is evident that there is some fault in the way researches on the subject have been hitherto conducted.

A malarial subject, being considered as a soil for the culture of germs, we should seek whether his organism is favorable or not to the proliferation of the tuberculous microbe; in other words, we should try to determine whether a subject exempt from other disease than malarial poisoning, resists better infection by the tuberculous germ, or becomes more easily its prey. Reciprocally, a tuberculous patient being taken as soil of culture, it will be necessary to determine, in a malarial region, what is his receptivity for the germ of malaria.

It may be, perhaps, at a future day possible to demonstrate: 1st. That soil already sown with paludism (malaria) is not proper for the development of the tuberculous granule, which would prove the correctness of Boudin's opinion. 2d. That on the contrary, the phthisical subject attacked by intermittent fever presents a graver form of the malady and a greater predisposition to pernicious attacks.

As we cannot actually sow and cultivate the microbe in the human subject, the field of experimentation on animals has been largely extended; it is however, necessary to properly choose soil, favorable or unfavorable, for the culture of the different morbid germs.

These different soils are furnished by the diverse species of animals which sometimes represent with more or less fidelity the principal diatheses of the human species. In order to study, for instance, the points relative to the microbe of gangrenous affections, neither the dog nor cat should be chosen, but the sheep or cow. To cultivate the tuberculous germ, there is no organism prepared by scrofula, but the monkey or calf should be taken in preference to the horse or pig.

Like individuals in a cachectic condition, who are almost without defence against most parasitic diseases, there are certain animals, rabbits, for instance, who receive and permit the development of all germs, from those of septicæmia to those of tuberculosis, as also the bacterides of malignant charbon. And just as there are certain individuals, who inhabit most unhealthy localities and are exposed to the most fatal epidemics without contracting any disease, even the most contagious, in like manner there are certain animals, the pig, for instance, who remain refractory to all forms of inoculation.—*Verneuil, Prof. of Surgery of the Paris Faculty, in the Med. and Surg. Reporter.*

